# Life Cycle Plasticity of *Colpoda aspera* Fed With Petroleum Tolerant Gram‐Positive and Gram‐Negative Bacteria

**DOI:** 10.1111/jeu.70085

**Published:** 2026-05-05

**Authors:** Laura Mondragón‐Camarillo, Cruz Carlos Castillo Camacho, Salvador Rodríguez‐Zaragoza

**Affiliations:** ^1^ Laboratorio Ecología Microbiana UBIPRO, FES‐Iztacala UNAM Tlalnepantla de Baz México

## Abstract

Soil recovery after contamination relies on the surviving microbiota to reconstruct microbial food webs. The ciliate *Colpoda aspera* and *Brevundimonas* sp., *Rhizobium* sp1, *Rhizobium* sp2 (gram‐negative), *Bacillus* sp1, *Bacillus* sp2, and *Microbacterium* sp.(gram‐positive) remain active after pulses of light petroleum contamination. We used phase contrast microscopy to assess excystation time and cyst production by *C. aspera* after placing 65 cysts in petri dishes with 0.5% LNA medium and adding 100 μL of a 5.2 × 10^8^ cell bacterial inoculum from contaminated soil. *C. aspera* excystation was monitored by triplicate every hour for 72 h. Excystation occurred faster and resulted in a higher number of cysts when fed with gram‐negative bacteria, particularly *Rhizobium* sp2 and *Brevundimonas* sp. In contrast, *C. aspera* cysts formed only two daughter cells when fed with *Microbacterium* sp., reducing the quantity and speed of reproduction. This reproductive behavior may allow other predatory ciliates to coexist when *Microbacterium* sp. becomes prevalent in a soil hotspot. Ciliates' feeding preferences and nutritional value are required to understand resource partitioning and understanding these interactions can inform strategies to promote resilient microbial communities and accelerate recovery after contamination.

## 1 Introduction

Soil recovery following contamination depends on the surviving microbiota for contaminant metabolization and microbial food web restoration to support plant establishment and productivity (Mondragón‐Camarillo et al. 2020; Jia et al. 2023). Plant productivity relies on growth‐promoting bacteria that offer assimilable forms of nitrogen, phosphorous, iron, and several other metabolites (Rodríguez and Fraga 1999; Egamberdiyeva and Höflich 2004; Berg 2009; Naqqash et al. 2020; Hawxhurst et al. 2023). Plants rely indirectly on bacterivorous organisms releasing nutrients trapped in bacterial biomass, which produces humified organic matter and improves soil fertility (Clarholm 1985; Griffiths 1986; Rodríguez and Fraga 1999; Egamberdiyeva and Höflich 2004; Berg 2009; Naqqash et al. 2020; Hawxhurst et al. 2023). Among bacterivorous organisms, ciliates respond faster to bacteria relative to other bacterivorous organisms. Ciliates' predatory activity stimulates bacterial growth, which creates a positive feedback loop by releasing essential nutrients, such as nitrogen and phosphorus, in forms readily available to plants (Bamforth 1995; Acosta‐Mercado and Lynn 2004; Hines et al. 2020; Martínez‐Reyes et al. 2022). Protist bacterivores connect microbial producers with higher levels of soil food webs, forming a strong link between the microbiome and soil mesofauna (Foissner 2014; Jousset et al. 2017; Asiloglu et al. 2021). Overall, plant growth and soil fertility depend on the soil food web structure, including bacterial and fungal decomposers (Bamforth 1995; Acosta‐Mercado and Lynn 2004; Hines et al. 2020).

Bacterivorous ciliates exhibit a degree of selectivity toward bacterial species—ranging from those that may feed on almost any bacteria, including the pigmented ones, to specialists that feed on a reduced number of bacterial species (Foissner 2016). Rapid increased bacterial growth is often followed by a surge in the growth of ciliates feeding on them. Ciliates typically form cysts when water is limited, and a smaller number of active bacteria attach to the jelly surrounding their cysts. The relevance of protist species in the soil system is correlated to its cyst abundance (Bamforth 2001; Foissner 2016; Hines et al. 2020; Li et al. 2024). The family Colpodidae accounts for about 55% of active and resting ciliates in soil (Bamforth 2001) and the genus *Colpoda* is notably abundant in various soils, including forests and cultivated areas, dominating in soils contaminated with hydrocarbons and heavy metals (Campbell et al. 1997; Bamforth 2001; Li et al. 2010; Mondragón‐Camarillo et al. 2020). *Colpoda aspera* forms both resistance and reproductive cysts in its life cycle and is one of the few ciliates that remain active after exposure to light crude oil (Mondragón‐Camarillo et al. 2020). Following the active consumption of bacteria, trophozoites form tetragenic cysts that produce four daughter cells. Additionally, resistant cysts form when food is limited or in face of other adverse events. The encystation process has been widely documented (Yamasaki et al. 2004; Müller et al. 2010; Matsuoka 2021); however, *Colpoda*'s activation process has been rarely reported. We observed that *C. aspera* cysts from oil‐contaminated soil always had bacterial strains attached to their jelly, making it impossible to preserve *C. aspera* axenically. We aimed to determine if these bacterial species attached to cysts can trigger *C. aspera* excystation and if the bacterial species affect cyst production, especially since several of them are known to promote plant growth.

## 2 Material and Methods

### 2.1 Isolation and Culture Conditions of *C. aspera*

*C. aspera* was isolated from soil contaminated with 50,000 ppm light crude oil. LNA (tryptone 10 g, yeast extract 5 g, NaCl 5 g) culture medium 0.5% was used to grow *C. aspera* until encystation, which generally occurs after 7 days. The cysts were concentrated by centrifugation at a low velocity (3000 rpm) and kept in a washing solution (1 mM Tris–HCl, pH 7.2, 100 μg/mL of Amikacin) for 3 h (Funadani et al. 2016). Bacteria adhered to cysts' gel were washed out by centrifuging in a tween solution (0.1 mM CaCl_2_ 1% [*w*/*v*]), Tween 80% (NP‐40), and 1 mM Tris–HCl (pH 7.2) twice to ensure that bacteria‐free cysts of *C. aspera* were obtained.

Three strains of gram‐positive and three strains of gram‐negative bacteria were isolated from the same contaminated soil and kept in LNA agar plates. The strains were initially separated by differences in colony morphology and then isolated and harvested for 16S rDNA identification. Bacterial DNA was extracted following the Doyle and Doyle (1987) protocol, and the DNA was purified by running in 1% agarose gel electroporation at 100 V for 30 min. Thereafter, universal primers FD1 (5′ AGAGTTTGATCCTGGCTCAG 3′) and RD1 (5′ AAGGAGGTGATCCAGCC 3′) (Weisburg et al. 1991) were used for amplification of the 16S rDNA. The PCR mixture for each strain was set up by using 50 ng of template DNA, 0.2 μM of dNTP, 2.5 mM MgCl_2_, and 1.25 U Taq polymerase in PCR buffer adjusted for a final volume of 50 μL. Subsequently, the Thermo‐Scientific Piko Thermal Cycler was set up for the initial step (5 min, 95°C), followed by 25 cycles of denaturing (30 s, 95°C), annealing (40 s, 57°C), and polymerization (2 min, 72°C). A final step (5 min, 72°C) was carried out. All PCR products were electrophoresed at 100 V for 30 min in Gel Extraction Kit and Clean UP (PureDirex) in accordance with the manufacturer instructions. These products were sequenced at Macrogen company (Seoul, South Korea). Then, sequences were edited with the BioEdit program and compared with those found in NCBI‐BLAST. *C*. *aspera* was identified by morphology alone (Foissner 1993).

### 2.2 Experimental Setup

Each bacterial strain was grown in 0.5% LNA medium. Bacterial concentration was measured using colorimetry, with 100 μL of culture medium inoculated into 20 mL fresh medium and adjusted to a final concentration of 5.2 × 10^8^ bacteria/mL. Thereafter, 65 cysts of *C. aspera* were inoculated and observed after 1 h under a phase contrast microscope (Nikon Eclipse 80i) at 40× to observe excystation. Subsequently, cultures were observed every hour for 6 h to determine the excystation time. Bacterial concentrations were determined 14, 18, 24, 48, and 72 h after the first *C. aspera* excystation. This procedure was repeated thrice for each bacterial strain.

Cysts of *C*. *aspera* produced in the presence of each bacterial strain were quantified after 72 h of incubation at 24°C by counting cysts in 1 mL of medium using a phase‐contrast microscope at 40× magnification. These data were used to calculate the generational time of *C. aspera* with every bacterial strain by using the Vater–Dobberstein and Hilfrich formula (Petz et al. 1985):

$$g=\left(\log N_{2}-\log N_{1}\right)/\text{logc}\ldots r=g/t\ldots d=1/r$$

where *g* denotes the number of divisions; *N*_1_ and *N*_2_ denote the number of cells at *t*_1_ and *t*_2_, respectively; *c* denotes the “birth” rate by cyst; *r* denotes the division rate by time unit; *t* denotes the time between counts (*t*_2_ − *t*_1_); and *d* denotes the generational time.

### 2.3 Statistical Analysis

A one‐way ANOVA was performed to evaluate generational time and cyst production in *C. aspera* . Additionally, an independent sample *t*‐test was conducted to compare excystation time between cells fed on gram‐negative and gram‐positive bacteria.

## 3 Results

The gram‐positive bacterial strains were identified to genus level as *Bacillus* sp1, *Bacillus* sp2, and *Microbacterium* sp., and the three gram‐negative bacteria corresponded to *Rhizobium* sp1, *Rhizobium* sp2, and *Brevundimonas* sp.

**FIGURE 1:**
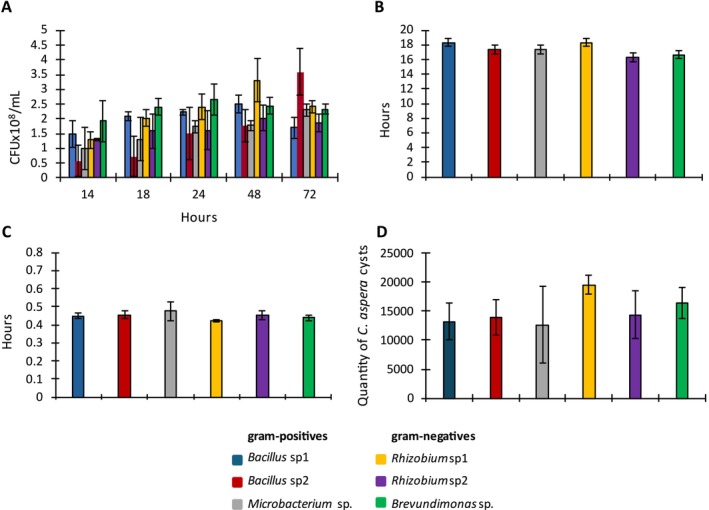
(A) Population size of six bacterial strains over 72 h of cultivation together with *C. aspera* . The initial population size at time zero (T0) for all strains were 5.2 × 10^8^ CFU/mL (colony‐forming units). Strains were quantified at 14, 18, 24, 48, and 72 h after *C. aspera's* excystation. (B) Excystation time (hours) of *C. aspera* associated with each bacterial strain. (C) Generation time of *C. aspera* (hours) when fed with the different bacterial strains. (D) Cyst productivity of *C. aspera* after feeding on each of the six bacterial strains. *C. aspera* produced a higher number of resting cysts when fed with gram‐negative bacteria. Data are means of three replicates, with error bars indicating standard deviation.

*Brevundimonas* sp. exhibited the greatest population growth after 14 h of cocultivation with *C. aspera* , whereas *Bacillus* sp2 had the lowest. At 48 h, gram‐negative bacteria had reached their maximum growth, with *Rhizobium* sp1 being the most abundant. In contrast, the gram‐positive bacteria *Bacillus* sp2 and *Microbacterium* sp. continued to increase to 72 h, while all other strains declined from their 48‐h levels (Figure 1A).

*C. aspera* excysted faster in the presence of gram‐negative bacteria, such as *Rhizobium* sp2 and *Brevundimonas* sp., although there was no statistical difference (*p* < 0.207) in excystation time between these strains (approximately 16 h). In contrast, excystation was slower with *Bacillus* sp1 and *Rhizobium* sp1 (18 h), and intermediate with *Bacillus* sp2 and *Microbacterium* sp. (17 h; Figure 1B).

The generational time of *C. aspera* was shorter (*p* < 0.32) when fed with *Rhizobium* sp1 (0.42 ± 0.01 h) and *Brevundimonas* sp. (0.44 ± 0.01 h) but was longer (*p* < 0.32) with *Microbacterium* sp. (0.47 ± 0.05 h; Figure 1C).

*C. aspera* produced a greater number of cysts on gram‐negative bacteria compared to gram‐positive bacteria; however, the difference was not significant (*p* < 0.063). *C. aspera* produced more cysts when fed with *Rhizobium* sp1, followed by *Brevundimonas* sp. and *Rhizobium* sp2 (Figure 1D).

**FIGURE 2:**
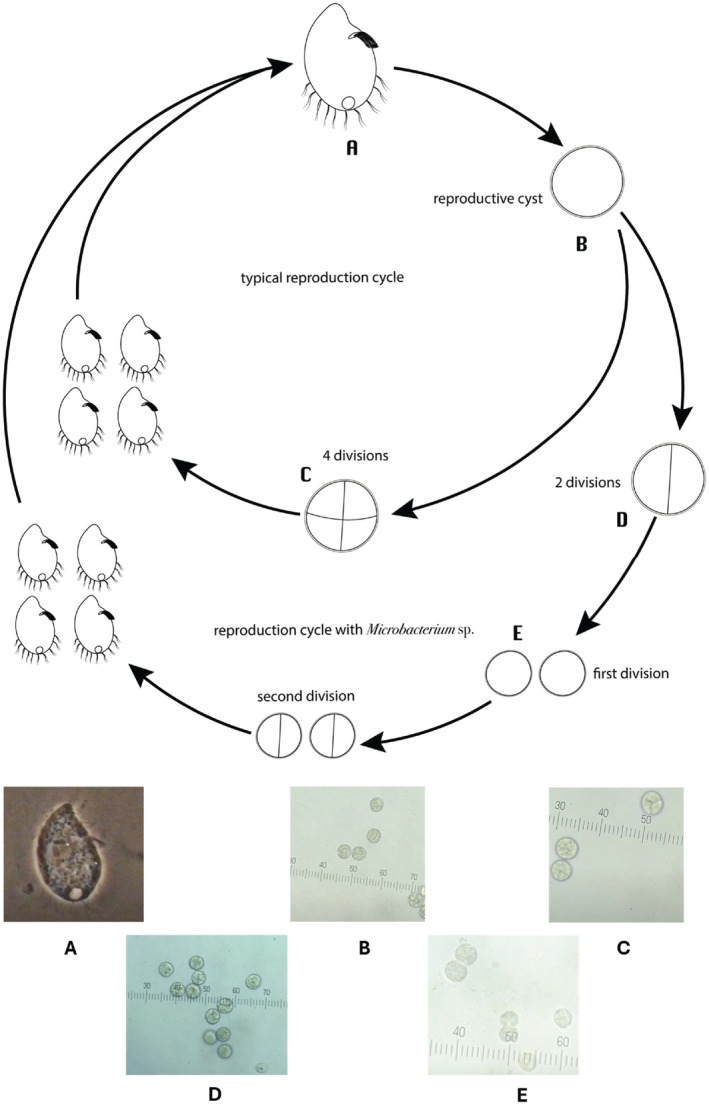
Life cycle *C. aspera* . (A) Trophic stage of *C. aspera* feeding actively on bacteria; (B) reproductive cyst; (C) formation of a tetrad (four cells inside the reproductive cyst) typical of the genus *Colpoda*; (D) reproductive cysts of *C. aspera* after fed with *Microbacterium* sp., cysts only divide into two cells; (E) the two cells immediately start dividing to produce another two cells. Pictures taken with a phase contrast microscope (A) 100×, (B–F) 40×.

The life cycle of *C. aspera* showed two different morphologies of reproductive cysts depending on the bacterial strain provided as food. *C. aspera* showed the reproductive cysts with the typical four divisions (Figure 2A) when fed with *Bacillus* sp1, *Bacillus* sp2, *Brevundimonas* sp., *Rhizobium* sp1, and *Rhizobium* sp2, and once excysted, the four trophozoites immediately started feeding on bacteria. However, when feeding on *Microbacterium* sp., *C. aspera* generated reproductive digenic cysts in the first step (Figure 2B), and thereafter, two trophonts appeared, where each divided and produced two trophonts each (Figure 2C). Feeding on *Microbacterium* sp. produced four trophonts as well, but in two steps instead of one.

We observed that *C. aspera* saved the bacteria it was feeding on in the jelly around the cysts and temporarily inside the cell—a behavior that could be more frequent than previously thought.

## 4 Discussion

Ciliates respond to unfavorable soil conditions by forming cysts and enter a dormant state until soil conditions become favorable again. *Colpoda* species form reproductive cysts when feeding on actively growing bacteria. It is proposed that excystation is activated by peptides or proteins released by bacteria and sensed by protists (Yamasaki et al. 2004; Matsuoka 2021). There is insufficient information regarding the functioning of these signals and their specificity across different species. A significant variation exists between the signals of gram‐positive and gram‐negative bacteria, with colpodids showing quicker responses to gram‐negative bacteria. In this study, *Rhizobium* sp2 and *Brevundimonas* sp., which are both gram‐negative, elicited a faster excystation response in *C. aspera* . However, *Brevundimonas* sp. required higher population density than *Rhizobium* sp2 to induce *C. aspera* excystation, indicating that a larger signal concentration is necessary to reach the detection threshold of *C. aspera* cysts. Similarly, *C. aspera* required a higher population density of *Bacillus* sp1 (gram‐positive) and *Rhizobium* sp1 (gram‐negative) compared to that of *Brevundimonas* sp. to trigger excystation (Figure 1A,B). Furthermore, this process took a longer time, notwithstanding they had higher population densities than *Microbacterium* sp. and *Bacillus* sp2.

These variations indicate that bacteria generate a wide range of chemical signals, with each bacterial species potentially possessing a distinct set of signals for communication. This enables signal receptors to detect the presence of specific bacterial species within biofilms or around *C. aspera* cysts. Nevertheless, chemical signals are merely one aspect of the predation process performed by *C. aspera* ; additional conditions are required to initiate the excystation of these protists. Factors such as the production of other types of secondary metabolites, cell wall structure, and compounds unrelated to the Gram stain (Chandarana et al. 2022) must also be detected.

Mounting evidence indicates that protists exhibit a preference for gram‐negative bacteria over gram‐positive bacteria. For instance, *Tetrahymena pyriformis* has shown a notable affinity for *Xanthomonas retroflexus* and *Stenotrophomonas rhizophila* , both of which are gram‐negative (Raghupathi et al. 2018). Furthermore, ciliates such as *Tetrahymena* sp. and *Chilodonella* sp. can consume biofilms composed of either *Pseudomonas* sp. (gram‐negative) or *Serratia plymuthica* (gram‐positive). However, both species demonstrated a preference for biofilms containing only *Pseudomonas* sp., suggesting that these ciliates utilize chemical signals in water to locate and differentiate between gram‐negative and gram‐positive bacteria (Dopheide et al. 2011). Free‐living soil amoebae also display strong preferences for gram‐negative bacteria. In the case of *Dictiostellium discoideum*, the preference for *Pseudomonas fluorescens* has been attributed to the high levels of cAMP produced by the bacterium, which serves as a potent chemical attractant for amoebae (Rashidi and Ostrowski 2019).

Motility and cell size can influence protists' preferences for gram‐negative bacteria (Dopheide et al. 2011). *Colpoda aspera*, like other protists, exhibits a strong preference for gram‐negative bacteria and has shorter generational times when feeding exclusively on *Rhizobium* sp1 or *Brevundimonas* sp. (Figure 1C,D), indicating that *C. aspera* can obtain all nutrients essential for cell growth and reproduction from these bacteria. This substantiates the idea that these ciliates can endure rapid population growth of these bacteria almost immediately upon soil hydration, explaining their abundance in highly disturbed soils (Mondragón‐Camarillo et al. 2020). Gram‐negative bacteria had higher population growth rates than gram‐positive ones over 72 h, resulting in more bacterial biomass available for *C. aspera* , and consequently more cysts of this ciliate after the experimentation period. In natural systems, this predation may promote bacterial growth by freeing space and releasing nutrients fixed within bacterial biomass in biofilms (Risse‐Buhl et al. 2012).

The presence of bacteria inside protist cysts has been previously reported, such as in the genus *Dictyostelium* and *Acanthamoeba*. The ecological significance of these observations is still debated as amoebae may “seed” these bacteria as an additional food source and bacteria may even produce compounds protecting the amoebae from pathogens or competitors, conferring ecological advantages (DiSalvo et al. 2014; Shi et al. 2021). Bacteria frequently become trapped in the mucus around ciliate cysts, becoming the food source after ciliate excystation (Verni and Rosati 2011; Li et al. 2022). In the case of *C. aspera* , bacteria were observed both around and within the cyst. This phenomenon could reflect a strategy that favors the survival and persistence of both protists and their food source in adverse environmental conditions, suggesting a more complex and resilient ecological relationship between ciliates and bacteria in contaminated environments.

### 4.1 *C. aspera* Life Cycle

*C. aspera* , as in all other members of this genus, forms a tetragenic reproductive cyst that produces four daughter cells by asexual reproduction (Lüftenegger et al. 1985). On occasions, a digenic (two daughter cells) or an octogenic (eight daughter cells) cyst is formed (Padnos et al. 1954). While this variability was observed, no explanation was forwarded to explain why these variations happened. In our study, feeding *C. aspera* exclusively with each one of five bacterial species provided enough nutrients for the ciliate to produce tetragenic cysts. Feeding exclusively on the *Microbacterium* sp. (gram‐positive) produced a variation in the reproductive strategy of *C. aspera* . Instead of forming tetragenic cysts, it formed only digenic ones, where both daughter cells divided again, resulting in four daughter cells. We attributed this change in reproductive strategy to either poor digestion of eaten *Microbacterium* sp. or lack of nutrients in the *Microbacterium* cells. The nutrients or molecules missing in *Microbacterium* sp. may be critical for the formation of tetragenic cysts in a single step, leading to the production of only digenic cells in *C. aspera* and thus requiring two steps to fulfill the process. This alternate form of reproduction of *C. aspera* is less efficient than the more common, tetragenic form. Consequently, it produces fewer cysts with *Microbacterium* sp. than the ones produced with the other gram‐positive bacteria, although the number of cysts produced with *Microbacterium* sp. was not significantly lower (*p* > 0.94) from those produced by feeding on *Bacillus* sp1 and *Bacillus* sp2.

Ciliates can feed on various types of soil bacteria, resulting in a very diverse diet rather than a specialized one. By feeding on different bacterial species, *C. aspera* can obtain the variety of nutrients needed for reproduction. Soil contamination alters the composition of bacterial species, eliminating the sensitive and favoring resistant ones, depending on the severity of the disturbance. Contamination alters the species' composition, thereby reducing the diversity of protists that are essential for regenerating food webs. The significance of *C. aspera* in contaminated soils lies in its ability to tolerate contamination, consume thriving soil bacteria, and stimulate the restoration of microbial food webs through its predatory activities. By preying on bacteria, regardless of their digestibility or nutritional value, *C. aspera* adapts its mode of asexual reproduction and enhances its reproductive efficiency to endure contamination.

## 5 Conclusion

*C. aspera* formed cysts faster and showed a higher reproductive rate with the gram‐negative *Rhizobium* sp2 and *Brevundimonas* sp. compared to gram‐positive *Microbacterium* sp. or *Bacillus*. Chemical signals related to nutrient content of bacteria appeared to play a role as *C. aspera* required a higher population density of gram‐positive than of gram‐negative bacteria for excystation. *C. aspera* exhibited a variant of reproductive cysts, reducing its efficiency by producing two sets of two daughter cells instead of four when fed exclusively with *Microbacterium* sp., suggesting that this reproductive behavior may allow other ciliates to dominate when this bacterial genus becomes prevalent in a soil hotspot. Further understanding of ciliates' feeding preferences and the nutritional value of gram‐positive bacteria is needed to understand the resource partitioning between soil ciliates and the dynamics of microbial food webs during soil restoration.

## Supporting information


**Data S1:** Supporting Information on statistical analyses.

**Data S2:** Supporting Information. The origin and characteristics of soil.

**Video S1:** jeu70085‐sup‐0003‐VideoS1.mp4. *C. aspera* reproductive cyst. Formation of a tetrad (four cells inside the reproductive cyst) typical of the genus *Colpoda*.

**Video S2:** Reproductive cysts of *C. aspera* after fed with *Microbacterium* sp., cysts only divide into two cells.

**Data S3:** Sequences availability.

## Data Availability

The data that supports the findings of this study are available in the Supporting Information of this article.
