# Supplementary material for: Life Cycle Plasticity of Colpoda aspera Fed With Petroleum Tolerant Gram‐Positive and Gram‐Negative Bacteria
Source: J Eukaryot Microbiol. 2026 May 5;73(3):e70085. doi: 10.1111/jeu.70085 (PMC13140017; doi:10.1111/jeu.70085)
Supplement: Supplementary file 1 — Data S1: Supporting Information on statistical analyses. [file JEU-73-e70085-s003.docx]

1. **ANOVA Colony-Forming Units (CFU) of Bacterial**

**14 hours**

| Source | GL | SS | MS | F | Pr > F | p-values signification codes |
| --- | --- | --- | --- | --- | --- | --- |
| Model | 5.000 | 3.129 | 0.626 | 2.358 | 0.104 | ° |
| Error | 12.000 | 3.185 | 0.265 |  |  |  |
| Corrected total | 17.000 | 6.314 |  |  |  |  |
|  | | | | | | |
| *Signification codes: 0 < *** < 0.001 < ** < 0.01 < * < 0.05 < . < 0.1 < ° < 1* | | | | | | |
| **18 hours**   \| Source \| GL \| SS \| MS \| F \| Pr > F \| p-values signification codes \| \| --- \| --- \| --- \| --- \| --- \| --- \| --- \| \| Model \| 5.000 \| 5.609 \| 1.122 \| 4.298 \| **0.018** \| * \| \| Error \| 12.000 \| 3.132 \| 0.261 \|  \|  \|  \| \| Corrected total \| 17.000 \| 8.742 \|  \|  \|  \|  \| \|  \| \| \| \| \| \| \| \| *Signification codes: 0 < *** < 0.001 < ** < 0.01 < * < 0.05 < . < 0.1 < ° < 1* \| \| \| \| \| \| \|   **24 hours**   \| Source \| GL \| SS \| MS \| F \| Pr > F \| p-values signification codes \| \| --- \| --- \| --- \| --- \| --- \| --- \| --- \| \| Model \| 5.000 \| 3.367 \| 0.673 \| 2.342 \| 0.105 \| ° \| \| Error \| 12.000 \| 3.450 \| 0.288 \|  \|  \|  \| \| Corrected total \| 17.000 \| 6.818 \|  \|  \|  \|  \| \|  \| \| \| \| \| \| \| \| *Signification codes: 0 < *** < 0.001 < ** < 0.01 < * < 0.05 < . < 0.1 < ° < 1* \| \| \| \| \| \| \|   **48 hours**   \| Source \| GL \| SS \| MS \| F \| Pr > F \| p-values signification codes \| \| --- \| --- \| --- \| --- \| --- \| --- \| --- \| \| Model \| 5.000 \| 5.143 \| 1.029 \| 4.993 \| **0.011** \| * \| \| Error \| 12.000 \| 2.472 \| 0.206 \|  \|  \|  \| \| Corrected total \| 17.000 \| 7.616 \|  \|  \|  \|  \| \|  \| \| \| \| \| \| \| \| *Signification codes: 0 < *** < 0.001 < ** < 0.01 < * < 0.05 < . < 0.1 < ° < 1* \| \| \| \| \| \| \|   **72 hours**   \| Source \| GL \| SS \| MS \| F \| Pr > F \| p-values signification codes \| \| --- \| --- \| --- \| --- \| --- \| --- \| --- \| \| Model \| 5.000 \| 6.612 \| 1.322 \| 8.348 \| **0.001** \| ** \| \| Error \| 12.000 \| 1.901 \| 0.158 \|  \|  \|  \| \| Corrected total \| 17.000 \| 8.513 \|  \|  \|  \|  \| \|  \| \| \| \| \| \| \| \| *Signification codes: 0 < *** < 0.001 < ** < 0.01 < * < 0.05 < . < 0.1 < ° < 1* \| \| \| \| \| \| \|  1. **Excystation Time in *C*. *aspera***  \|  \| Excystation Time in C. aspera \| \| \|  \|  \|  \| \| --- \| --- \| --- \| --- \| --- \| --- \| --- \| \|  \| *Rhizobium* sp2 \| *Brevundimonas* sp. \| *Bacillus* sp2 \| *Microbacterium* sp. \| *Rhizobium* sp1 \| *Bacillus* sp1 \| \| hours \| 16 \| 16 \| 17 \| 17 \| 18 \| 18 \| \|  \| 16 \| 17 \| 17 \| 17 \| 18 \| 18 \| \|  \| 17 \| 17 \| 18 \| 18 \| 19 \| 19 \|   **t-Test of Excystation Time in *C*. *aspera***   \| t-Test: Two-Sample Assuming Equal Variances \| \| \| \| \| --- \| --- \| --- \| --- \| \|  \|  \|  \|  \| \|  \| *gram-negative* \| *gram-positive* \|  \| \| Mean \| 17.11111 \| 17.66667 \|  \| \| Variance \| 1.111111 \| 0.5 \|  \| \| Observations \| 9 \| 9 \|  \| \| Pooled Variance \| 0.805556 \|  \|  \| \| Hypothesized Mean Difference \| 0 \|  \|  \| \| df \| 16 \|  \|  \| \| t Stat \| -1.31306 \|  \|  \| \| P(T<=t) one-tail \| 0.103841 \|  \|  \| \| t Critical one-tail \| 1.745884 \|  \|  \| \| P(T<=t) two-tail \| 0.207682 \|  \|  \| \| t Critical two-tail \| 2.119905 \|  \|  \|  1. **ANOVA of the Number of Cysts in *C*. *aspera***  \| Anova: Single Factor \| \|  \|  \|  \|  \|  \| \| --- \| --- \| --- \| --- \| --- \| --- \| --- \| \|  \|  \|  \|  \|  \|  \|  \| \| SUMMARY \| \|  \|  \|  \|  \|  \| \| *Groups* \| *Count* \| *Sum* \| *Average* \| *Variance* \|  \|  \| \| gram-positive \| 9 \| 47798.4 \| 5310.933 \| 2549955 \|  \|  \| \| gram-negative \| 9 \| 60381.2 \| 6709.022 \| 1867416 \|  \|  \| \|  \|  \|  \|  \|  \|  \|  \| \|  \|  \|  \|  \|  \|  \|  \| \| ANOVA \|  \|  \|  \|  \|  \|  \| \| *Source of Variation* \| *SS* \| *df* \| *MS* \| *F* \| *P-value* \| *F crit* \| \| Between Groups \| 8795936 \| 1 \| 8795936 \| 3.982431 \| 0.063292 \| 4.493998 \| \| Within Groups \| 35338964 \| 16 \| 2208685 \|  \|  \|  \| \|  \|  \|  \|  \|  \|  \|  \| \| Total \| 44134901 \| 17 \|  \|  \|  \|  \|  1. **ANOVA of the Generational Time of *C. aspera***  \| SUMMARY \|  \|  \|  \|  \|  \|  \| \| --- \| --- \| --- \| --- \| --- \| --- \| --- \| \| *Groups* \| *Count* \| *Sum* \| *Average* \| *Variance* \|  \|  \| \| Bacillus sp 2 \| 3 \| 1.3666958 \| 0.45556527 \| 0.00049884 \|  \|  \| \| Microbacterium sp. \| 3 \| 1.42462373 \| 0.47487458 \| 0.00258703 \|  \|  \| \| Bacillus sp1 \| 3 \| 1.34656193 \| 0.44885398 \| 0.0002885 \|  \|  \| \| Rhizobium sp1 \| 3 \| 1.26372541 \| 0.4212418 \| 3.8426E-05 \|  \|  \| \| Rhizobium sp2 \| 3 \| 1.35708918 \| 0.45236306 \| 0.00072097 \|  \|  \| \| Brevundimonas sp. \| 3 \| 1.317662 \| 0.43922067 \| 0.00020685 \|  \|  \| \|  \|  \|  \|  \|  \|  \|  \| \|  \|  \|  \|  \|  \|  \|  \| \| ANOVA \|  \|  \|  \|  \|  \|  \| \| *Source of variations* \| *SS* \| *df* \| *MS* \| *F* \| *P- value* \| *F crit* \| \| Between groups \| 0.00476847 \| 5 \| 0.00095369 \| 1.31828447 \| 0.32029307 \| 3.10587524 \| \| Within groups \| 0.00868123 \| 12 \| 0.00072344 \|  \|  \|  \| \|  \|  \|  \|  \|  \|  \|  \| \| Total \| 0.01344971 \| 17 \|  \|  \|  \|  \| | | | | | | |
