# Supplementary material for: Life Cycle Plasticity of Colpoda aspera Fed With Petroleum Tolerant Gram‐Positive and Gram‐Negative Bacteria
Source: J Eukaryot Microbiol. 2026 May 5;73(3):e70085. doi: 10.1111/jeu.70085 (PMC13140017; doi:10.1111/jeu.70085)
Supplement: Supplementary file 2 — Data S2: Supporting Information. The origin and characteristics of soil. [file JEU-73-e70085-s005.docx]

Origin of the soil from which *C*. *aspera* and six bacterial strains were isolated

Soil used for microcosms preparation was collected from a cornfield in Tultitlán, Estado de Mexico, Mexico, which had no record of petroleum contamination. Following the removal of harvested plants, a composite soil sample was obtained from ten distinct sites within the field spaced 10 m apart. Samples were homogenized into a single volume, and the soil mixture had the following characteristics: clay loam texture (50% sand, 26% clay, 24% silt), with a pH 7.62, organic matter 7.99%, NH₄⁺ 3.16 mg kg⁻¹, NO₃⁻ 2.05 mg kg⁻¹, and available phosphorus 4.63 mg kg⁻¹ (Mondragon-Camarillo et al. 2020).

Petroleum used in this study was light crude oil (Isthmus, API 32), obtained through a donation from the Tula refinery. Soil contamination was achieved by gradually adding petroleum to the soil, followed by mechanical mixing until a homogeneous distribution was achieved, with a final concentration of 50 g/kg. The mixture was then left to stand for 24 h to allow evaporation of volatile compounds. Finally, the soil moisture was adjusted to 60% of the soil's field capacity using sterile distilled water (Mondragon-Camarillo et al. 2020).

Mondragón-Camarillo, L., S. Rodríguez-Zaragoza, M.R. Mendoza-López, N. Cabirol, and M. Macek. 2020. “Recovery of Soil Protozoan Community Structure Promoted by *M*. *sativa* After a Strong Pulse of Hydrocarbon Contamination.” Water, Air, and Soil Pollution 231 (6): 1–15. https://doi.org/10.1007/s11270-020-04618-7
