# Supplementary material for: Life Cycle Plasticity of Colpoda aspera Fed With Petroleum Tolerant Gram‐Positive and Gram‐Negative Bacteria
Source: J Eukaryot Microbiol. 2026 May 5;73(3):e70085. doi: 10.1111/jeu.70085 (PMC13140017; doi:10.1111/jeu.70085)
Supplement: Supplementary file 5 — Data S3: Sequences availability. [file JEU-73-e70085-s002.docx]

*Rhizobium* sp. strain sp1 16S ribosomal RNA gene, partial sequence. GenBank: PX461597. (GenBank submission: SUB15713875).

*Rhizobium* sp. strain sp2 16S ribosomal RNA gene, partial sequence. GenBank: PX457881. (GenBank submission: SUB15690755).

*Bacillus* sp. strain sp1 16S ribosomal RNA gene, partial sequence. GenBank: PX461602. (GenBank submission: SUB15713927).

*Bacillus* sp. strain sp2 16S ribosomal RNA gene, partial sequence. GenBank: PX461614. (GenBank submission: SUB15713967).

*Brevundimonas* sp. strain sp1 16S ribosomal RNA gene, partial sequence. GenBank: PX461663. (GenBank submission: SUB15690755).

*Microbacterium* sp. strain sp1 16S ribosomal RNA gene, partial sequence. GenBank: PX461668. (GenBank submission: SUB15714151).
